# Plasma Heparin-Binding Protein as a Predictor of Functional Recovery and a Potential Therapeutic Target in Acute Anterior Circulation Large-Vessel Occlusion Stroke

**DOI:** 10.3390/brainsci15111216

**Published:** 2025-11-12

**Authors:** Chao Wu, Hedi An, You Yin, Dongya Huang

**Affiliations:** Department of Neurology, Shanghai East Hospital, School of Medicine, Tongji University, Shanghai 200120, China; 2180274@tongji.edu.cn (C.W.); anhedi2025@163.com (H.A.); yinyou@tongji.edu.cn (Y.Y.)

**Keywords:** heparin-binding protein (HBP), mechanical thrombectomy (MT), anterior circulation large-vessel occlusion (AC-LVO), acute ischemic stroke, prognosis, neuroinflammation, prognostic biomarker

## Abstract

**Background:** Patients with acute anterior circulation large-vessel occlusion (AC-LVO) stroke frequently experience poor outcomes despite successful mechanical thrombectomy (MT). Heparin-binding protein (HBP), a neutrophil-derived mediator of inflammation and vascular permeability, may contribute to neuroinflammation and prognosis; however, its role in stroke remains unclear. **Methods:** In this retrospective study, 163 patients with AC-LVO stroke who underwent MT were enrolled. Plasma HBP levels were measured within 24 h after thrombectomy. Functional outcomes were evaluated using the modified Rankin Scale (mRS) at 90 days, with an mRS score 3–6 suggesting a poor outcome. Multivariable logistic regression and receiver operating characteristic (ROC) analyses were performed to assess associations between HBP and outcomes. **Results:** Of the 163 patients, 88 (54.0%) had poor functional outcomes. The median plasma HBP level of patients with poor outcomes was significantly higher than that of patients with good outcomes (28.80 vs. 18.42 ng/mL; *p* < 0.001). HBP remained independently associated with poor outcome (odds ratio [OR] 1.04; 95% confidence interval [CI] 1.01–1.07; *p* = 0.002) after adjusting for demographic, clinical, procedural, and laboratory covariates. ROC analysis showed a modest predictive value of HBP alone (area under the curve [AUC] = 0.671), whereas adding HBP to clinical models improved prognostic accuracy (AUC = 0.835 for model 2; AUC = 0.889 for model 3). **Conclusions:** For patients with AC-LVO stroke, elevated plasma HBP within 24 h after MT serves as an independent predictor of unfavorable functional outcomes at 90 days. Thus, HBP may serve as a prognostic biomarker and potential target for immunomodulatory therapy.

## 1. Introduction

Acute anterior circulation large-vessel occlusion (AC-LVO) stroke is the leading cause of mortality and long-term disability worldwide, accounting for a substantial proportion of the 12.2 million new cases of stroke and 6.55 million stroke-related deaths annually [1]. This subtype of ischemic stroke, caused by large-vessel occlusion in the anterior circulation, is often severe and associated with functional dependence, cognitive impairment, and post-stroke complications, such as pneumonia and hemorrhagic transformation. Although mechanical thrombectomy (MT) significantly improves recanalization rates and functional outcomes in selected patients, a substantial proportion fails to achieve favorable recovery despite successful reperfusion [2,3], suggesting that mechanisms beyond vascular occlusion—particularly neuroinflammation—contribute significantly to neurological injury.

Neuroinflammation in cerebral ischemia–reperfusion injury has gained increasing attention in recent years [4,5]. Following infarction, ischemic brain tissue releases damage-associated molecular patterns (DAMPs), which activate microglia and astrocytes, triggering a local inflammatory cascade [6,7]. Neutrophils, the first immune cells to infiltrate, aggravate blood–brain barrier disruption and neuronal injury by releasing reactive oxygen species, proteases, and proinflammatory cytokines, such as interleukin-6 (IL-6) and tumor necrosis factor-α (TNF-α) [8,9]. Over the past decade, numerous studies have identified the association of elevated C-reactive protein (CRP) levels with poor outcomes, mortality, and hemorrhagic transformation (HT) [10,11], while neutrophil-to-lymphocyte ratio (NLR) and platelet-to-lymphocyte ratio (PLR) are correlated with neurological deterioration, post-stroke pneumonia, and unfavorable functional outcomes [12,13].

Heparin-binding protein (HBP), a protein released predominantly by neutrophils, is a key biomarker in infectious diseases, including sepsis and coronavirus disease 2019 (COVID-19) [14,15]. HBP promotes vascular permeability and disrupts endothelial integrity by inducing cytoskeletal rearrangement and opening endothelial junctions [16,17], thereby compromising the blood–brain barrier (BBB)—a critical event in cerebral ischemia, leading to edema, hemorrhagic transformation, and neuronal damage. Recent studies suggest that HBP may also predict disease severity and prognosis in cardiovascular disorders, including acute myocardial infarction, atherosclerosis, and myocarditis [18,19]. However, its role in non-infectious inflammatory conditions, such as cerebral infarction, particularly in the specific context of patients with AC-LVO treated with MT, remains insufficiently explored and poorly defined.

This study aimed to evaluate the association between early plasma HBP levels and 90-day functional outcomes in patients with AC-LVO stroke undergoing MT to provide a rationale for risk stratification, individualized therapy, and inflammation-targeted interventions in AIS.

## 2. Materials and Methods

### 2.1. Patient Population

We conducted a single-center, retrospective, observational cohort study of consecutive patients with AIS due to AC-LVO who underwent MT at Shanghai East Hospital, Affiliated with Tongji University, China, between April 2022 and June 2025. This study adhered to the Declaration of Helsinki and obtained approval from the Medical Ethics Committee of Shanghai East Hospital (Approval No.: 200120).

Eligible patients were required to meet the following inclusion criteria: age ≥ 18 years; time from symptom onset to treatment initiation within 6 h, or 6–24 h if additional clinical or imaging criteria (e.g., DAWN or DEFUSE-3) were satisfied; AIS with AC-LVO, diagnosed based on computed tomography angiography (CTA); baseline National Institutes of Health Stroke Scale (NIHSS) score ≥6 at admission; pre-stroke mRS score ≤2; and provision of informed consent by the patient or a legally authorized representative.

**Figure 1:**
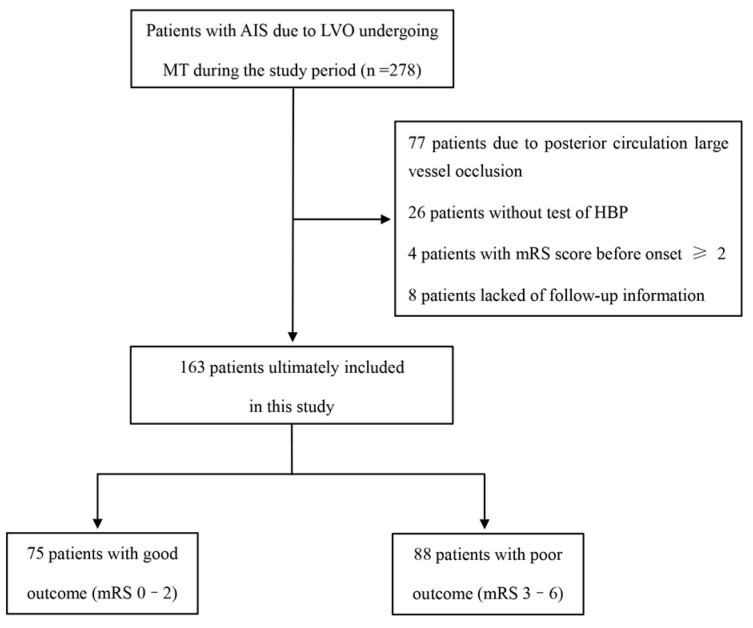
Flow chart of patient selection. Among 278 patients screened initially, exclusions were made for posterior circulation occlusions, missing HBP testing, pre-stroke mRS ≥ 2, or loss to follow-up, leaving 163 patients for analysis. Abbreviations: AIS, acute ischemic stroke; MT, mechanical thrombectomy; LVO, large-vessel occlusion; HBP, heparin-binding protein; mRS, modified Rankin Scale.

Exclusion criteria were as follows: presence of intracranial hemorrhage (ICH) or bleeding tendency confirmed by CT; CT imaging revealed either early ischemic changes exceeding one-third of the MCA territory or Alberta Stroke Program Early CT Score (ASPECTS) < 6; severe concomitant systemic disease, such as cardiac, pulmonary, hepatic, or renal failure, or other life-threatening illness; life expectancy <6 months, history of severe psychiatric illness interfering with neurological evaluation, or presence of brain tumors or cerebral arteriovenous malformations; and incomplete baseline data or failure to obtain plasma HBP testing within 24 h after MT. The patient selection process is summarized in Figure 1.

A prospective sample size was calculated assuming that the proportion of patients with elevated HBP levels would be at least 10% higher in the poor functional outcome group (mRS 3–6) than in the good outcome group (mRS 0–2). With a two-sided type-I error (α) of 0.05 and a power (1 − β) of 80%, the minimum sample size required for each cohort was 105.

### 2.2. Data Collection

Clinical, demographic, and laboratory data were collected from patient medical records. Demographic variables included age and sex, and cerebrovascular risk factors comprised hypertension, diabetes mellitus, atrial fibrillation, smoking history, and prior transient ischemic attack (TIA) or stroke. Clinical characteristics at admission included baseline NIHSS score, random blood glucose, and HbA1c. Stroke etiology was classified according to the Trial of ORG 10172 in Acute Stroke Treatment (TOAST) criteria, and the site of vascular occlusion was identified using CTA. Treatment-related variables included onset-to-door time, onset-to-puncture time, puncture-to-recanalization time, and whether intravenous thrombolysis was performed. Plasma HBP levels were measured using a commercial HBP assay kit (Immunofluorescence Dry Quantitative Method) manufactured by Joinstar Biotechnology Co., Ltd. (Hangzhou, China). Blood samples were collected within 24 h after successful recanalization, and all samples were drawn in the fasting state. Laboratory analyses encompassed complete blood count, lipid profile (TC, TG, HDL, LDL), glucose metabolism markers (FBG, HbA1c), along with D-dimer, HCY, HBP, CRP, SAA, NLR, LMR, and PLR.

### 2.3. Clinical Outcomes

The primary endpoint was the functional outcome at 90 days, assessed using the mRS. A favorable functional outcome was defined as an mRS score of 0–2, indicating functional independence, whereas a score of 3–6 was classified as a poor outcome. Secondary endpoints included HT and symptomatic intracranial hemorrhage (sICH). sICH was defined according to the Heidelberg criteria as intracranial hemorrhage on post-procedural imaging associated with an increase of ≥4 points in the NIHSS score or death within 24 h after MT.

### 2.4. Statistical Analyses

Data are presented as frequencies with percentages for categorical variables. Continuous data are summarized as mean ± standard deviation for normally distributed variables or median with interquartile range for non-normally distributed variables. Group comparisons were performed using independent-samples *t*-tests for normal data, the Mann–Whitney U test for non-normal data, and Pearson’s chi-square or Fisher’s exact test for categorical variables, as appropriate.

Multivariable logistic regression analysis was conducted to identify independent predictors of poor outcomes. Multicollinearity was assessed using the variance inflation factor (VIF) and was confirmed as absent (all VIFs < 5). Three progressively adjusted models were constructed: Model 1 was unadjusted; Model 2 was adjusted for demographic and clinical factors (age, cardiovascular disease, baseline NIHSS score, large-artery atherosclerosis, and HT); and Model 3 was additionally adjusted for procedural and laboratory parameters, including neutrophil count, D-dimer, HCY, CRP, SAA, HBP, and HbA1c.

The predictive performance was evaluated by receiver operating characteristic (ROC) curve analysis. To internally validate the models and mitigate overfitting, a 10-fold cross-validation was performed using a random split of the data. Furthermore, the net reclassification improvement (NRI) and integrated discrimination improvement (IDI) were calculated to quantify the incremental predictive value of HBP over established inflammatory biomarkers. All analyses were conducted using SPSS 22.0 (IBM Corporation, Armonk, NY, USA) and R software (version 4.5.1; R Foundation for Statistical Computing, Vienna, Austria). A two-sided *p*-value < 0.05 defined statistical significance.

## 3. Results

### 3.1. Demographic and Clinical Characteristics

A total of 278 patients with LVO-AIS underwent MT during the study period. After excluding patients with posterior circulation occlusions (n = 77), without HBP testing (n = 26), with pre-stroke mRS ≥ 2 (n = 4), or without follow-up data (n = 8), 163 patients were included in the final analysis. Among these, 88 (54.0%) had poor functional outcomes (mRS 3–6) at 90 days (Figure 1).

Baseline demographic and clinical characteristics are presented in Table 1. Patients with poor outcomes were significantly older (median 75 vs. 71 years, *p* = 0.036) and had higher baseline NIHSS scores (median 15.83 vs. 11.99, *p* < 0.001). TOAST classification showed that large-artery atherosclerosis was less frequent in the poor-outcome group compared with the favorable-outcome group (36.4% vs. 56.0%, *p* = 0.012). HT (44.3% vs. 14.7%, *p* < 0.001) and sICH (28.4% vs. 9.3%, *p* = 0.002) were more common among patients with poor outcomes.

No significant differences were observed between groups with respect to sex distribution, smoking status, vascular risk factors (diabetes mellitus, hypertension, previous TIA or stroke, cardiovascular disease, atrial fibrillation), baseline glucose, site of occlusion, or procedural characteristics.

### 3.2. Laboratory Findings

**Figure 2:**
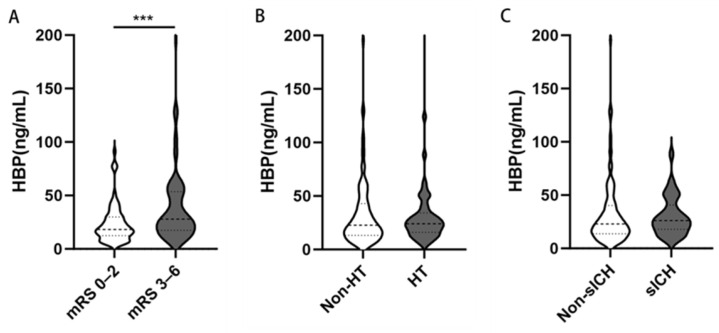
Distribution of plasma HBP according to clinical outcomes in patients with LVO-AIS treated with MT. Violin plots show HBP levels stratified by (**A**) 90-day functional outcome, (**B**) HT, and (**C**) sICH. Medians (horizontal lines), interquartile ranges (boxes), ranges (whiskers), and individual data points are shown. Patients with poor outcomes had significantly higher HBP levels compared with those with favorable outcomes (28.80 vs. 18.42 ng/mL, *p* < 0.001). No significant differences were observed between groups with and without HT or sICH. *** *p* < 0.001. Abbreviations: HBP, heparin-binding protein; mRS, modified Rankin Scale; LVO, large-vessel occlusion; AIS, acute ischemic stroke; HT, hemorrhagic transformation; sICH, symptomatic intracranial hemorrhage; MT, mechanical thrombectomy.

Patients with poor outcomes exhibited significantly higher plasma HBP levels than those with favorable outcomes (28.80 vs. 18.42 ng/mL, *p* < 0.001; Table 2; Figure 2A). However, no significant differences in HBP levels were observed between groups with and without HT or sICH (Figure 2B,C).

As summarized in Table 2, patients with poor outcomes exhibited evidence of heightened inflammatory activity, including elevated white blood cell counts (9.57 vs. 8.07 × 10^9^/L, *p* < 0.001), neutrophil counts (8.29 vs. 6.53 × 10^9^/L, *p* < 0.001), monocyte counts (0.48 vs. 0.41 × 10^9^/L, *p* < 0.001), NLR (10.76 vs. 6.82, *p* < 0.001), LMR (1.61 vs. 2.38, *p* < 0.001), and PLR (228 vs. 202, *p* = 0.045). Inflammatory cytokines showed marked differences, with higher CRP (13.15 vs. 7.22 mg/L, *p* = 0.004), SAA (31.92 vs. 8.43 mg/L, *p* < 0.001), and HBP (28.80 vs. 18.42 ng/mL, *p* < 0.001) in the poor-outcome group.

Metabolic parameters were altered, with elevated homocysteine (15.00 vs. 11.30 µmol/L, *p* < 0.001) and D-dimer (1.80 vs. 1.09 mg/L, *p* = 0.002) observed in patients with poor outcomes. Other laboratory indicators showed no significant differences between groups.

### 3.3. Associations of HBP with Poor Outcomes

**Figure 3:**
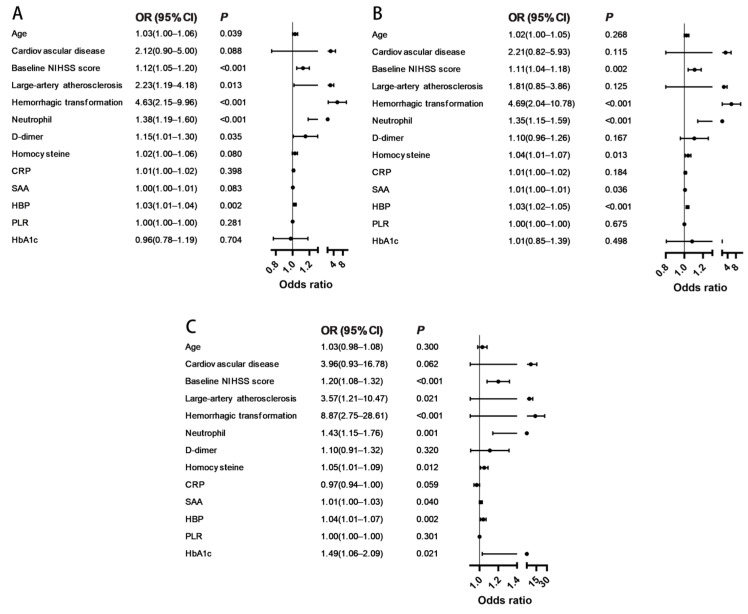
Forest plots of univariable and multivariable logistic regression analyses. The analysis presents adjusted ORs and 95% CIs. (**A**) Model 1, unadjusted; (**B**) Model 2, adjusted for demographic and clinical variables; (**C**) Model 3, fully adjusted for demographic, clinical, procedural, and laboratory parameters. Abbreviations: NIHSS, National Institutes of Health Stroke Scale; SAA, serum amyloid A; CRP, C-reactive protein; HBP, heparin-binding protein; PLR, platelet-to-lymphocyte ratio; OR, odds ratio; CI, confidence interval.

To assess the robustness of the association between HBP and functional outcomes, three progressively adjusted logistic regression models were constructed. In univariable analysis (Model 1), age, baseline NIHSS score, large-artery atherosclerosis, HT, neutrophil count, D-dimer, HCY, CRP, SAA, and HBP were all significantly associated with outcome after MT (Table 3; Figure 3A).

After adjusting for demographic and clinical factors (age, cardiovascular disease, baseline NIHSS score, large-artery atherosclerosis, and HT) in Model 2, HBP remained independently associated with outcome (odds ratio [OR] 1.03; 95% confidence interval [CI] 1.02–1.05; *p* < 0.001; Table 3; Figure 3B). In the fully adjusted Model 3, which additionally included neutrophil count, D-dimer, HCY, CRP, SAA, PLR and HbA1c, HBP continued to show a significant association with poor outcome (OR 1.04; 95% CI 1.01–1.07; *p* = 0.002; Table 3; Figure 3C).

To validate our findings across the full spectrum of disability, an ordinal logistic regression analysis was performed using the 90-day mRS (0–6). This analysis confirmed that higher HBP levels were independently associated with worse functional outcomes (OR 1.02; 95% CI 1.01–1.03; *p* < 0.001; Supplementary Table S1).

### 3.4. Comparative and Predictive Value of HBP for Poor 90-Day Functional Outcomes

**Figure 4:**
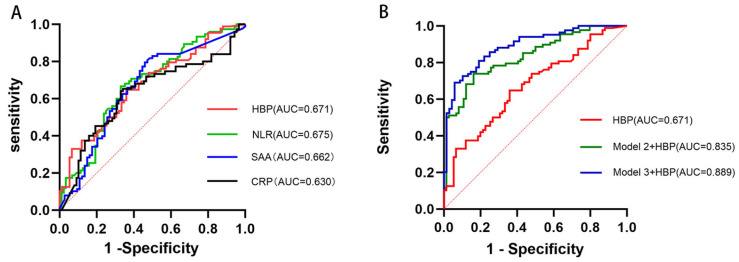
ROC curves for the prediction of 90-day functional outcomes. (**A**) ROC curves of individual biomarkers (HBP, NLR, SAA, CRP) for predicting 90-day functional outcomes. (**B**) ROC curves comparing HBP alone to the baseline (Model 2) and fully adjusted (Model 3) clinical models, demonstrating enhanced predictive performance when HBP is integrated into the models. Abbreviations: AUC, area under the curve; ROC, receiver operating characteristic; SAA, serum amyloid A; CRP, C-reactive protein; NLR, neutrophil-to-lymphocyte ratio; HBP, heparin-binding protein.

HBP alone demonstrated modest prognostic ability, with an AUC of 0.671 (95% CI, 0.59–0.75; *p* < 0.001), an optimal cutoff value of 22.89 ng/mL, sensitivity of 64.8%, and specificity of 64.0%. The AUC of HBP was not significantly superior to that of NLR (0.675), SAA (0.662), or CRP (0.630) in direct comparison (DeLong’s test, all *p* > 0.05) (Figure 4A). Therefore, we investigated its incremental value beyond these markers and standard clinical models. Incorporation of HBP into the baseline clinical model (Model 2) significantly improved predictive performance, with an AUC of 0.835 (95% CI, 0.77–0.90; *p* < 0.001). Addition of HBP to the fully adjusted model (Model 3) enhanced prognostic accuracy, yielding the highest AUC of 0.889 (95% CI, 0.84–0.94; *p* < 0.001) (Figure 4B).

To address potential overfitting and rigorously assess the generalizability of our models, we performed 10-fold cross-validation. The results confirmed the robust predictive performance of the model incorporating HBP, clinical, and laboratory parameters (Model 3), which retained a high cross-validated AUC of 0.844 (95% CI: 0.794–0.894). The minimal decrease from the original AUC (0.899) indicates a stable and reliable model (Table 4).

### 3.5. Assessment of Model Improvement and Overfitting for Model 3

To quantify the incremental predictive value of the fully adjusted model, we calculated the net reclassification improvement (NRI) and integrated discrimination improvement (IDI). The analysis yielded a categorical NRI of 0.1615 (95% CI: 0.04–0.28; *p* = 0.007), indicating significantly improved risk stratification. Furthermore, the IDI was 0.1578 (95% CI: 0.10–0.21; *p* < 0.001), confirming a substantial improvement in the model’s overall discriminatory power.

## 4. Discussion

This study demonstrates that elevated plasma HBP levels are significantly associated with poor 90-day functional outcomes in patients with AC-LVO stroke undergoing MT. While prior studies have consistently shown the association of CRP and NLR with worse outcomes after MT [10,13], our data suggest that HBP may capture a distinct and more specific aspect of neutrophil-mediated injury. This is the first study to identify HBP as an independent prognostic biomarker for functional outcomes in this specific population treated with endovascular therapy.

Patients with poor outcomes (mRS 3–6) had significantly higher plasma HBP levels compared with those with favorable outcomes (mRS 0–2). Importantly, this association remained robust after progressive adjustment for confounders across three models. In the fully adjusted model (Model 3), which accounted for demographic, clinical, procedural, and laboratory parameters—including neutrophil count and other inflammatory markers such as CRP and SAA—HBP was an independent predictor of poor outcomes (OR 1.04; 95% CI, 1.01–1.07; *p* = 0.002). These findings suggest that the prognostic value of HBP extends beyond reflecting neutrophil burden and is not fully explained by other systemic inflammatory markers.

ROC curve analysis highlighted the potential clinical utility of HBP. Although HBP alone showed only modest discriminative ability (AUC = 0.671), incorporation into clinical models markedly enhanced prognostic accuracy. Adding HBP to the baseline clinical model (Model 2) increased the AUC to 0.835, and inclusion in the fully adjusted model (Model 3) yielded the highest predictive accuracy (AUC = 0.889). These results indicate that HBP provides prognostic information complementary to established clinical and inflammatory risk factors, suggesting its potential use in refining risk stratification in patients who achieve successful recanalization.

While CRP, SAA, and NLR are systemic inflammatory markers, HBP is a direct mediator of vascular injury. Known functions of HBP support the biological plausibility of these findings. HBP, released predominantly from activated neutrophils, is a potent inducer of vascular permeability and a key mediator of systemic inflammation [17,20]. In AIS, reperfusion after recanalization may trigger ischemia–reperfusion injury characterized by intense neuroinflammatory activation [21]. Neutrophil infiltration into reperfused tissue likely drives substantial HBP release, which in turn exacerbates BBB disruption, promotes cerebral edema, facilitates HT, and amplifies local inflammatory cascades [22,23]. These processes contribute to adverse outcomes despite technically successful vascular recanalization, a phenomenon often referred to as “futile recanalization” [24,25].

We found that elevated HBP is associated with poor outcomes. By disrupting endothelial tight junctions, HBP directly compromises blood–brain barrier integrity, thereby promoting cerebral edema and hemorrhagic complications. This extends the clinical significance of HBP beyond infectious diseases like sepsis [26,27] to the non-infectious neuroinflammation of acute ischemic stroke. Furthermore, emerging evidence suggests a potential novel mechanism whereby HBP-induced vascular leakage may be mediated by activating the TGF-β/Smad signaling pathway [23]. This proposed pathway, however, remains speculative in the setting of cerebral ischemia and warrants direct experimental validation in future stroke models. Given its pivotal role in driving these detrimental processes, HBP represents a promising potential target for future immunomodulatory therapies aimed at mitigating reperfusion injury and improving functional outcomes after thrombectomy.

Our findings should be interpreted considering the limitations of this study. First, the single-center, retrospective design may have introduced selection bias. Second, we could not fully account for potential confounders that might influence HBP levels, such as concurrent infection, contrast-induced inflammation, or the use of anticoagulant therapy. Third, plasma HBP levels were measured only once, within 24 h after admission; dynamic monitoring of HBP during and after MT might provide additional insights into the temporal profile of neuroinflammation and its association with outcomes [28].

Future prospective multicenter studies with serial HBP measurements are needed to validate these findings and clarify the kinetics of HBP release following MT. Most importantly, our results illuminate HBP as a potential therapeutic target. Consequently, future experimental studies should directly investigate the efficacy of HBP-targeted interventions, such as neutralizing monoclonal antibodies or small-molecule inhibitors, to mitigate reperfusion injury and improve recovery after thrombectomy. Furthermore, exploring the combination of HBP with advanced neuroimaging biomarkers (e.g., assessing blood–brain barrier permeability or ischemic penumbra profiles) may yield a superior, multi-modal model for individualized prognostication and tailored post-procedural care.

## 5. Conclusions

This study provides novel evidence that elevated plasma HBP is an independent predictor of poor 90-day functional outcome in patients with AC-LVO stroke treated with MT. Incorporation of HBP into clinical models significantly improved prognostic accuracy, underscoring the contribution of neutrophil-driven inflammation to post-stroke recovery. These findings highlight HBP as both a promising biomarker for individualized risk stratification and a potential target for future immunomodulatory interventions.

## Figures and Tables

**Table 1:** Baseline characteristics by 90-day functional outcome.

	90-Day Functional Outcome			
Variables	Overall n = 163	mRS 0–2 n = 75	mRS 3–6 n = 88	*p* Value
**Baseline characteristics**				
Age, years	73 (66–79)	71 (65.75–80.75)	75 (68.75–82.25)	0.036
Sex, Men, n (%)	114 (69.9)	50 (66.7)	64 (72.7)	0.400
Current smoker, n (%)	60 (36.8)	28 (17.2)	32 (19.6)	0.898
**Medical history, n (%)**				
Hypertension	109 (66.9)	49 (65.3)	60 (68.2)	0.700
Diabetes mellitus	50 (30.7)	22 (29.3)	28 (31.8)	0.732
Previous TIA/stroke	36 (22.1)	14 (18.7)	22 (25.0)	0.331
Cardiovascular disease,	26 (16.0)	16 (21.3)	10 (11.4)	0.083
Atrial fibrillation	5 1(31.3)	16 (21.3)	35 (39.8)	0.110
**Initial clinical assessment**				
Baseline NIHSS score	14.07 ± 6.32	11.99 ± 5.54	15.83 ± 6.42	<0.001
Baseline glucose, mmol/L, median (IQR)	7.20 (6.30–9.25)	6.50 (5.83–7.78)	7.70 (6.40–10.38)	0.100
**Stroke etiology, n (%)**				
Large-artery atherosclerosis	74 (45.4)	42 (56.0)	32 (36.4)	0.012
Cardioembolism	75 (46.0)	28 (37.3)	47 (53.4)	0.400
Others	14 (8.6)	5 (6.7)	9 (10.2)	0.419
**Site of occlusion [n, (%)]**				
Distal ICA, M1, M2 occlusion	124 (76.1)	62 (82.7)	62 (70.5)	
Tandem occlusion	39 (23.9)	13 (17.3)	26 (29.5)	0.069
**Procedural characteristics, n (%)**				
Intravenous thrombolysis	41 (25.2)	19 (25.3)	22 (25.0)	0.961
Unknown symptom onset, n (%)	58 (35.6)	26 (34.7)	32 (36.4)	0.822
Onset-to-door, min, median (IQR)	180 (120–300)	180 (120–293)	225 (180–300)	0.385
Onset-to-groin, mean (SD)	419.33 ± 165.99	402.62 ± 178.51	434.25 ± 154.06	0.267
Procedure duration, min, median (IQR)	110 (80–140)	105 (83–140)	115 (85–146)	0.158
**Clinical outcomes, n (%)**				
HT	50 (30.7)	11 (14.7)	39 (44.3)	<0.001
sICH	32 (19.6)	7 (9.3)	25 (28.4)	0.002

Note: Values are presented as mean ± SD, median with IQR, or number (%), as appropriate. Abbreviations: mRS, modified Rankin Scale; TIA, transient ischemic attacks; ICA, internal carotid artery; HT, Hemorrhagic transformation; sICH, symptomatic intracranial hemorrhage; NIHSS, National Institutes of Health Stroke Scale; IQR, interquartile range; SD, standard deviation.

**Table 2:** Comparison of laboratory findings between patients with favorable (mRS 0–2) and poor (mRS 3–6) 90-day outcomes.

Variables	90-Day Functional Outcome			
	Overall n = 163	mRS 0–2 n = 75	mRS 3–6 n = 88	*p*-Value
**Hematological parameters**				
White blood cell, ×10^9^/L, median (IQR)	9.00 (7.46–10.76)	8.07 (6.48–9.91)	9.57 (8.35–12.18)	<0.001
Neutrophil, ×10^9^/L, median (IQR)	7.49 (5.94–9.51)	6.53 (4.81–8.00)	8.29 (6.76–10.76)	<0.001
Lymphocyte, ×10^9^/L, median (IQR)	0.81 (0.60–1.25)	0.86 (0.61–1.43)	0.82 (0.59–1.08)	0.323
Monocyte, ×10^9^/L, median (IQR)	0.46 (0.33–0.62)	0.41 (0.23–0.58)	0.48 (0.37–0.65)	<0.001
Red blood cell, ×10^12^/L, mean (SD)	4.03 ± 0.62	4.04 ± 0.61	4.01 ± 0.64	0.74
Hemoglobin, g/L, median (IQR)	126(112–137)	127 (112–140)	125 (113–136)	0.494
Platelet count, ×10^9^/L, median (IQR)	174 (145–235)	171 (153–229)	179 (135–246)	0.757
**Biochemical markers**				
Triglycerides (mmol/L), median (IQR)	1.33 (0.93–1.99)	1.23 (0.83–1.94)	1.41 (0.99–2.07)	0.288
Total cholesterol (mmol/L), mean (SD)	3.81 ± 0.95	3.87 ± 0.90	3.76 ± 0.99	0.467
HDL (mmol/L), mean (SD)	1.06 ± 0.29	1.07 ± 0.33	1.06 ± 0.26	0.773
LDL (mmol/L), mean (SD)	2.25 ± 0.82	2.26 ± 0.82	2.24 ± 0.83	0.415
D-dimer(mg/L), median (IQR)	1.55 (0.75–3.85)	1.09 (0.57–2.55)	1.80 (0.95–4.22)	0.002
Homocysteine (µmol/L), median (IQR)	12.70 (9.40–19.50)	11.30 (8.65–14.85)	15.00 (10.80–21.40)	<0.001
HbA1c (mmol/mol), median (IQR)	6.00 (5.70–6.93)	5.90 (5.70–6.80)	6.20 (5.80–7.00)	0.839
**Inflammatory biomarkers**				
CRP (mg/L), median (IQR)	10.96 (4.40–23.13)	7.22 (2.74–15.91)	13.15 (6.32–23.74)	0.004
SAA (mg/L), median (IQR)	21.19 (8.01–59.42)	8.73 (8.00–32.07)	31.92 (11.47–75.38)	<0.001
HBP (ng/mL), median (IQR)	23.11 (14.78–40.25)	18.42 (12.03–30.84)	28.80 (18.48–53.94)	<0.001
NLR, median (IQR)	9.81 (5.41–13.48)	6.82 (4.92–10.67)	10.76 (6.41–15.94)	<0.001
LMR, median (IQR)	1.93 (1.31–3.12)	2.38 (1.70–4.00)	1.61 (1.1–2.40)	<0.001
PLR, median (IQR)	213 (148–316)	202 (137–290)	228 (168–356)	0.045

Values are presented as mean ± SD, or median with IQR. Abbreviations: HbA1c, glycated hemoglobin; HDL, high-density lipoprotein; LDL, low-density lipoprotein; CRP, C-reactive protein; SAA, serum amyloid A; HBP, heparin-binding protein; NLR, neutrophil-to-lymphocyte ratio; PLR, platelet-to-lymphocyte ratio; IQR, interquartile range; LMR, lymphocyte-to-monocyte ratio; SD, standard deviation; mRS, modified Rankin Scale.

**Table 3:** Logistic regression analysis of predictors of poor 90-day functional outcome (mRS 3–6). ORs and 95% CIs are presented for three progressively adjusted models.

Variables	Model 1		Model 2		Model 3	
	OR (95% CI)	*p*-Value	OR (95% CI)	*p*-Value	OR (95% CI)	*p*-Value
Age	1.03 (1.00–1.06)	0.039	1.02 (1.00–1.05)	0.268	1.03 (0.98–1.08)	0.300
Baseline NIHSS score	1.12 (1.05–1.20)	<0.001	1.11 (1.04–1.18)	0.002	1.20 (1.08–1.32)	<0.001
Large-artery atherosclerosis	2.23 (1.19–4.18)	0.013	1.81 (0.85–3.86)	0.125	3.57 (1.21–10.47)	0.021
Hemorrhagic transformation	4.63 (2.15–9.96)	<0.001	4.69 (2.04–10.78)	<0.001	8.87 (2.75–28.61)	<0.001
Neutrophil	1.38 (1.19–1.60)	<0.001	1.35 (1.15–1.59)	<0.001	1.43 (1.15–1.76)	0.001
D-dimer	1.15 (1.01–1.30)	0.035	1.10 (0.96–1.26)	0.167	1.10 (0.91–1.32)	0.320
Homocysteine	1.02 (1.00–1.06)	0.080	1.04 (1.01–1.07)	0.013	1.05 (1.01–1.09)	0.012
SAA	1.00 (1.00–1.01)	0.083	1.01 (1.00–1.01)	0.036	1.01 (1.00–1.03)	0.040
HBP	1.03 (1.01–1.04)	0.002	1.03 (1.02–1.05)	<0.001	1.04 (1.01–1.07)	0.002

Note: Model 1, unadjusted; Model 2, adjusted for age, cardiovascular disease, baseline NIHSS score, large-artery atherosclerosis, and hemorrhagic transformation; Model 3, additionally adjusted for laboratory parameters (neutrophil count, D-dimer, homocysteine, CRP, SAA, HBP, PLR and HbA1c). Abbreviations: NIHSS, National Institutes of Health Stroke Scale; SAA, serum amyloid A; CRP, C-reactive protein; HBP, heparin-binding protein; PLR, platelet-to-lymphocyte ratio; HbA1c, glycated hemoglobin; OR, odds ratio; CI, confidence interval; mRS, modified Rankin Scale.

**Table 4:** Model performance and internal validation via 10-Fold cross-validation.

Model	Original AUC (95% CI)	10-Fold Cross-Validation AUC (95% CI)
Model 1 (HBP)	0.671 (0.590–0.754)	0.690 (0.606–0.775)
Model 2 + HBP	0.835 (0.774–0.896)	0.819 (0.798–0.840)
Model 3 + HBP	0.889 (0.838–0.940)	0.844 (0.794–0.894)

## Data Availability

The fully de-identified dataset and analysis code generated during this study are not publicly available to guarantee patient privacy but will be made available from the corresponding author upon reasonable request. Requests can be submitted via email and will be promptly evaluated. A data sharing agreement may be required, and requestors will need to provide a scientifically sound research proposal.

## Supplementary Materials

The following supporting information can be downloaded at: https://www.mdpi.com/article/10.3390/brainsci15111216/s1, Figure S1: A histogram of the overall HBP levels for all patients, overlayed with a normal distribution curve. Figure S2: A Q-Q (Quantile-Quantile) plot for HBP. The substantial deviation of the data points from the straight reference line provides further graphical confirmation of the non-normal distribution. Table S1: Results of the Ordinal Logistic Regression Analysis for the 90-day modified Rankin Scale.

## Abbreviations

The following abbreviations are used in this manuscript:

AC-LVO	anterior circulation large-vessel occlusion
AIS	acute ischemic stroke
AUC	area under the curve
CI	confidence interval
CRP	C-reactive protein
CTA	computed tomography angiography
FBG	fasting blood glucose
HbA1c	glycated hemoglobin
HBP	heparin-binding protein
HDL	high-density lipoprotein
HCY	homocysteine
HT	hemorrhagic transformation
ICH	intracranial hemorrhage
ICA	internal carotid artery
IQR	interquartile range
IL-6	interleukin-6
LMR	lymphocyte-to-monocyte ratio
LDL	low-density lipoprotein
LVO	large-vessel occlusion
MCA	middle cerebral artery
mRS	modified Rankin Scale
MT	mechanical thrombectomy
NLR	neutrophil-to-lymphocyte ratio
NIHSS	National Institutes of Health Stroke Scale
OR	odds ratio
PLR	platelet-to-lymphocyte ratio
ROC	receiver operating characteristic
SAA	serum amyloid A
sICH	symptomatic intracranial hemorrhage
SD	standard deviation
TIA	transient ischemic attack
TG	triglycerides
TNF-α	tumor necrosis factor-alpha
TOAST	Trial of ORG 10172 in Acute Stroke Treatment
TC	total cholesterol
