# Supplementary material for: Plasma Heparin-Binding Protein as a Predictor of Functional Recovery and a Potential Therapeutic Target in Acute Anterior Circulation Large-Vessel Occlusion Stroke
Source: Brain Sci. 2025 Nov 12;15(11):1216. doi: 10.3390/brainsci15111216 (PMC12650104; doi:10.3390/brainsci15111216)
Supplement: Supplementary file 1 [file brainsci-15-01216-s001.zip › brainsci-3928127-supplementary.pdf]

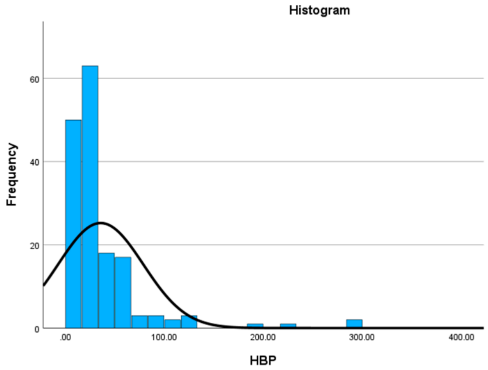

Supplementary Figure S1. A histogram of the overall HBP levels for all patients, overlayed with a normal distribution curve. This figure clearly shows the positive skewness of the HBP data.

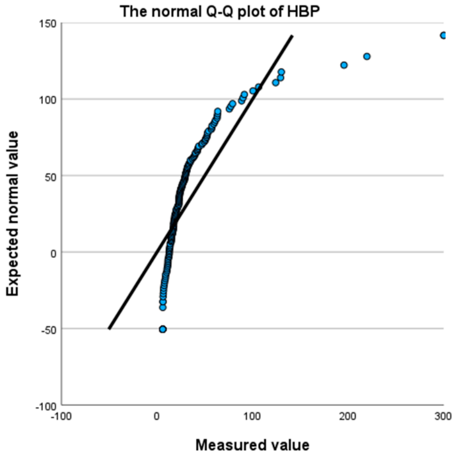

Supplementary Figure S2. A Q-Q (Quantile-Quantile) plot for HBP. The substantial deviation of the data points from the straight reference line provides further graphical confirmation of the non-normal distribution.

Supplementary Table S1. Results of the Ordinal Logistic Regression Analysis for the 90-day modified Rankin Scale.

**Variables**	**OR (95% CI)**	***p*-value**
Age	1.02(0.99–1.05)	0.267
Baseline NIHSS score	1.12 (1.06–1.18)	<.001
Large-artery atherosclerosis	0.79(0.41–1.51)	0.478
Hemorrhagic transformation	2.80(1.45–5.40)	0.002
Neutrophil	1.05(0.97–1.14)	0.203
D-dimer	1.05(0.95–1.17)	0.308
Homocysteine	1.02(1.00–1.04)	0.038
SAA	1.00(1.00–1.01)	0.375
HBP	1.02(1.01–1.03)	<.001

Abbreviations: OR, Odds Ratio; CI, Confidence Interval; NIHSS, National Institutes of Health Stroke Scale; SAA, Serum Amyloid A; HBP, Heparin-Binding Protein.
